# Prior Antibiotic Therapy and the Onset of Healthcare-Associated Infections Sustained by Multidrug-Resistant *Klebsiella pneumoniae* in Intensive Care Unit Patients: A Nested Case–Control Study

**DOI:** 10.3390/antibiotics10030302

**Published:** 2021-03-15

**Authors:** Giuseppe Migliara, Valentina Baccolini, Claudia Isonne, Sara Cianfanelli, Carolina Di Paolo, Annamaria Mele, Lorenza Lia, Angelo Nardi, Carla Salerno, Susanna Caminada, Vittoria Cammalleri, Francesco Alessandri, Guglielmo Tellan, Giancarlo Ceccarelli, Mario Venditti, Francesco Pugliese, Carolina Marzuillo, Corrado De Vito, Maria De Giusti, Paolo Villari

**Affiliations:** 1 Department of Public Health and Infectious Diseases, Sapienza University of Rome, 00185 Rome, Italy; valentina.baccolini@uniroma1.it (V.B.); claudia.isonne@uniroma1.it (C.I.); sara.cianfanelli@uniroma1.it (S.C.); carolina.dipaolo@uniroma1.it (C.D.P.); annamaria.mele@uniroma1.it (A.M.); lorenza.lia@uniroma1.it (L.L.); angelo.nardi@uniroma1.it (A.N.); carla.salerno@uniroma1.it (C.S.); susanna.caminada@uniroma1.it (S.C.); vittoria.cammalleri@uniroma1.it (V.C.); giancarlo.ceccarelli@uniroma1.it (G.C.); mario.venditti@uniroma1.it (M.V.); carolina.marzuillo@uniroma1.it (C.M.); corrado.devito@uniroma1.it (C.D.V.); maria.degiusti@uniroma1.it (M.D.G.); paolo.villari@uniroma1.it (P.V.); 2 Department of Anesthesia and Intensive Care Medicine Azienda, Universitaria-Ospedaliera Policlinico Umberto I, 00185 Rome, Italy; francesco.alessandri@uniroma1.it (F.A.); guglielmo.tellan@uniroma1.it (G.T.); f.pugliese@uniroma1.it (F.P.); 3 Department of Internal, Anesthesiological and Cardiovascular Clinical Sciences, Sapienza University of Rome, 00185 Rome, Italy; 4 Department of General and Specialist Surgery “P. Stefanini”, Sapienza University of Rome, 00185 Rome, Italy

**Keywords:** healthcare-associated infections, antibiotic exposure, intensive care unit, multidrug resistance, *Klebsiella pneumoniae*

## Abstract

Epidemiological research has demonstrated direct relationships between antibiotic consumption and the emergence of multidrug-resistant (MDR) bacteria. In this nested case–control study, we assessed whether prior exposure to antibiotic therapy and its duration affect the onset of healthcare-associated infections (HAIs) sustained by MDR *Klebsiella pneumoniae* (MDR-Kp) in intensive care unit patients. Cases were defined as patients who developed an MDR-Kp HAI. Controls matched on sex and the length of intensive care unit (ICU) stay were randomly selected from the at-risk population. Any antibiotic agent received in systemic administration before the onset of infection was considered as antibiotic exposure. Multivariable conditional logistic regression analyses were performed to estimate the effect of prior exposure to each antibiotic class (Model 1) or its duration (Model 2) on the onset of HAIs sustained by MDR-Kp. Overall, 87 cases and 261 gender-matched controls were compared. In Model 1, aminoglycosides and linezolid independently increased the likelihood of developing an MDR-Kp HAI, whereas exposure to both linezolid and penicillins reduced the effect of linezolid alone. In Model 2, cumulative exposure to aminoglycosides increased the likelihood of the outcome, as well as cumulative exposures to penicillins and colistin, while a previous exposure to both penicillins and colistin reduced the influence of the two antibiotic classes alone. Our study confirms that aminoglycosides, penicillins, linezolid, and colistin may play a role in favoring the infections sustained by MDR-Kp. However, several double exposures in the time window before HAI onset seemed to hinder the selective pressure exerted by individual agents.

## 1. Introduction

Healthcare-associated infections (HAIs) are a major patient safety issue in hospitals, especially in intensive care units (ICUs), leading to prolonged hospital stays and high morbidity and mortality rates and adding to the economic burden on healthcare systems [1,2]. The newly released third Extended Surveillance on Prevalence of Infection in Intensive Care study pointed out that among patients hospitalized in ICUs worldwide, more than half have developed at least one suspected or proven infection with a higher risk of death compared with community-acquired infections [3]. As for the microorganisms responsible for such infections, the most frequently isolated were Gram-negative species, especially *Klebsiella* species, *Escherichia coli, Pseudomonas aeruginosa*, and *Acinetobacter baumannii* complex species [3], followed by Gram-positive isolates, among which the most commonly detected was *Staphylococcus aureus* (7.4% methicillin-sensitive *S. Aureus*; 4.7% *S. Aureus* resistant to methicillin, linezolid, or vancomycin; and 4.6% methicillin-resistant *S. Aureus*) [3].

Additionally, hospital infections sustained by multidrug-resistant (MDR) bacterial organisms are increasing [4], with Gram-positive multidrug-resistant pathogens being overtaken by Gram-negative strain infections [5]. The burden of antimicrobial resistance is such that in 2019, more than half of the *E. coli* isolates and more than a third of the *K. pneumoniae* and *Acinetobacter* species isolates in Europe were resistant to at least one antimicrobial group and presented a combined resistance to several antimicrobial groups in 28.6% and 48.6% of the isolates, respectively [4]. Similar concerns apply to the United States, where in 2017, the proportions of resistant isolates in inpatients were an estimated 12.6%, 6.6%, and 1.2% for *Enterobacteriaceae* with extended-spectrum beta-lactamase (ESBL), multidrug-resistant, and carbapenem-nonsusceptible phenotypes, respectively, and 42.4% and 34.5% for *Acinetobacter* spp. with multidrug resistance and carbapeneme-nonsusceptible phenotypes, respectively [6]. For these reasons, carbapenem-resistant *A. baumannii* and carbapenem-resistant and/or third-generation cephalosporin-resistant *Enterobacteriaceae*, mostly represented by *K. pneumoniae*, are listed as critical pathogens on the World Health Organization priority list for effective drug development [7].

Although with different approaches [8], epidemiological research has demonstrated direct relationships between antibiotic consumption and the emergence of resistant strains [9,10,11]. For example, previous exposure to several antibiotic classes has been reported in the literature as a risk factor for hospital infections sustained by multidrug-resistant *K. pneumoniae* (MDR-Kp) [12,13,14] or carbapenem-resistant *Enterobacteriacae* [15], often yielding inconsistent results [16]. However, given that the antibiotic selective pressure is a key driver of antimicrobial resistance growth [9], their optimal use is crucial to reduce further the emergence, selection, and spread of multidrug-resistant strains and their consequences, in terms of both patients’ outcomes and healthcare costs [17,18,19]. Hence, additional efforts to assess the association between prior antibiotic exposure and the likelihood of infections sustained by resistant pathogens might be useful to guide healthcare professionals in the optimal choice of empiric antibiotic therapy [20]. In this study, we assessed whether prior exposure to antibiotic therapy and its duration affect the onset of HAIs sustained by MDR-Kp in ICU patients.

## 2. Results

### 2.1. Description of Cases and Controls

A total of 948 patients were enrolled in the surveillance system from 1st May 2016 to 30th September 2019. During this period, 87 patients were diagnosed with an HAI sustained by MDR-Kp and were, therefore, identified as cases. These patients were compared with 261 gender-matched patients without such HAIs (controls).

Clinical characteristics of the study population are reported in Table 1, with the statistical significance of the univariate test. Except for chronic kidney disease, which was slightly more prevalent among cases (8.1% vs. 3.1%, *p* = 0.048), no other characteristic was found different between the two groups.

Most patients were men (70.1%), with a mean age of 57.5 ± 17.3 years among the cases and 56.5 ± 18.4 years among the controls (*p* = 0.657). The main reason for admission to the ICU was a medical condition for both groups (49.4% of the cases and 47.5% of the controls). Overall, 32 (36.8%) cases and 80 (30.7%) controls had at least one pre-existing medical condition (*p* = 0.292), and diabetes mellitus was the most prevalent comorbidity in both groups (19.5% among the cases and 21.0% among the controls, *p* = 0.879).

The average Simplified Acute Physiology Score II (SAPS II) at ICU admission was similar among cases and controls (42.8 ± 13.7 vs. 40.6 ± 14.1, *p* = 0.195). The same applies to the average use of a central venous catheter (23.7 ± 17.6 days vs. 22.7 ± 17.8 days, *p* = 0.649), mechanical ventilation (23.3 ± 17.7 days vs. 21.7 ± 17.0 days, *p* = 0.442), and a urinary catheter (19.7 ± 13.4 days vs. 18.8 ± 16.3 days, *p* = 0.642). For a complete description of the clinical characteristic of the patients, please refer to Table 1.

As for prior antibiotic exposure, the proportion of patients with dichotomous exposure ranged from 96.6–92.7% for carbapenems to 25.3–19.2% for lipopeptides. Similarly, the antibiotic class with higher cumulative exposure was carbapenems (33.3 ± 36.5 days for the cases and 29.6 ± 34.3 days for the controls). Univariate analysis showed a significant difference between cases and controls only for aminoglycoside exposure coded as binary (yes vs. no) (27.6% vs. 15.7%, *p* = 0.014). Detailed prior dichotomic and cumulative exposure for each antibiotic class, with the corresponding *p*-value of the univariate test, is reported in Table 2.

Of the 87 HAIs sustained by MDR-Kp among the cases, 35 (40.2%) were multidrug resistant, 37 (42.5%) extensively drug resistant, and 15 (17.2%) pandrug resistant. Furthermore, 76 (87.3%) were ESBL positive and carbapenem resistant, 9 (10.3%) ESBL positive only, 1 (1.2%) carbapenem resistant only, and 1 (1.2%) neither ESBL positive nor carbapenem resistant.

Regarding the site of infection, 31 HAIs were primary bloodstream infections (BSIs, 35.6%), 28 were catheter-associated urinary tract infections (CAUTIs, 32.2%), 13 were ventilator-associated pneumonia (VAP) (15.0%), 11 were catheter-related BSIs (CRBSIs, 12.6%), and 4 were surgical site infections (SSIs, 4.6%) (Table 3). As for the 261 controls, at the time of selection, 32 (12.3%) had an infection sustained by microorganisms other than MDR-Kp (11 VAP, 8 CAUTIs, 8 BSIs, 4 CRBSIs, and 1 SSI), whereas 229 patients (87.7%) did not have any (Table 3). Specifically, eight infections (25%) of the controls were caused by *Acinetobacter baumannii*, four infections (12.5%) by *Pseudomonas aeruginosa*, four infections (12.5%) by non-MDR-Kp, four infections (12.5%) by *Staphylococcus aureus*, and the remaining 12 infections (37.5%) by other microorganisms (data not shown).

### 2.2. Multivariable Analyses

At multivariable analysis, patients previously exposed to aminoglycosides had an increased likelihood of developing an HAI sustained by MDR-Kp (odds ratio (OR) = 2.27; 95% CI = 1.08–4.76) (Table 4, Model 1). Similar findings were observed for prior dichotomous exposure to linezolid (OR = 4.64, 95% CI = 1.32–16.27) (Table 4, Model 1). However, prior dichotomous exposure to both linezolid and penicillins decreased the odds of the outcome exerted by linezolid alone (OR = 0.22, 95% CI = 0.05–0.88) (Table 3, Model 1). As for the other antibiotic classes investigated, carbapenems, extended-spectrum cephalosporins, glycopeptides, daptomycin, penicillins, and colistin did not seem to be independent risk factors for HAI onset (Table 4, Model 1). Similarly, patient age, type of admission to the ICU, SAPS II, exposure to mechanical ventilation, preexisting comorbidities, and year of admission did not affect the development of infections sustained by MDR-Kp (Table 4, Model 1).

Multivariable analysis using exposures to antibiotic classes and the cumulative days of therapy confirmed the previous model for aminoglycosides, as for each one-day increase in exposure, there was a higher likelihood of HAI onset sustained by MDR-Kp (OR = 1.15, 95% CI = 1.03–1.29) (Table 4, Model 2). Moreover, cumulative exposures to penicillins and colistin were found to independently increase the odds of the outcome (OR = 1.08, 95% CI = 1.01–1.16 and OR = 1.07, 95% CI = 1.02–1.14, respectively) (Table 4, Model 2). However, double exposure to penicillins and colistin reduced the likelihood conferred by the two antibiotic classes alone (OR = 0.996, 95% CI = 0.993–0.999) (Table 4, Model 2). Conversely, none of the other antibiotic classes that were studied seemed to affect HAI onset with MDR-Kp (i.e., carbapenems, extended-spectrum cephalosporins, glycopeptides, daptomycin, and linezolid) (Table 4, Model 2). Lastly, patient age, type of admission to the ICU, SAPS II, exposure to mechanical ventilation, preexisting comorbidities, and year of admission yielded results comparable with those obtained in Model 1, and none of them was found to be an independent risk factor (Table 4, Model 2).

## 3. Discussion

The expansion of multidrug-resistant bacteria is one of the main concerns for critically ill patients [21], and selecting an appropriate antibiotic regimen is essential to preventing the onset of multidrug-resistant infections [22]. In this study, we found that previous exposure to aminoglycosides, linezolid, penicillins, and colistin increases the likelihood of developing an infection sustained by MDR-Kp among ICU patients, contributing to strengthening the existing literature in this field, which is often inconsistent [15]. We also reported potentially beneficial interactions between penicillins and oxazolidinones or polymyxins, which, in our opinion, is worth further investigation. Hence, given these findings and the association between infections sustained by MDR-Kp and the increase in morbidity and mortality rates [23], optimal antibiotic use is crucial to reducing the emergence of such infections [20].

However, studying the effect of prior antibiotic exposure on infection sustained by drug-resistant bacteria poses significant methodological challenges, and consequentially, a variety of study designs have been developed to address such topics [24], each of which has its advantages and limitations [25]. The case–control study is an efficient method for investigating risk factors of a disease, including an infection sustained by drug-resistant microorganisms [24]. In this context, the selection of controls depends on the research question being asked [25]: when the authors are interested in identifying antibiotic classes that may favor an infection sustained by a specific antibiotic-resistant pathogen, the controls should be patients uninfected with the resistant organism of interest [25]. A few authors have argued that this approach cannot accurately differentiate whether a risk factor is associated with an infection sustained by a pathogen in general or favors its resistant profile in particular [25,26], meaning that in this way, risk factors for both the general infection and the multidrug-resistant profile are identified. However, this design allows matching cases to controls, with all the resulting benefits and, more limited, drawbacks [25]. The case–case–control design and the case–control–control design are variations of the case–control study that are proposed to overcome its main limitation [26]. Nevertheless, they are not immune to pitfalls either: on the one hand, they draw conclusions on the causal effect of antibiotic exposure on bacterial resistance from a qualitative comparison between two analyses [26]; on the other hand, they make matching an impracticable approach, leading to potential residual confounding [25].

Given these considerations, we performed a classical case–control study for several reasons. First, our surveillance system registered only 14 cases of HAIs sustained by non-MDR-Kp, largely hindering the opportunity to apply the case–control–control and case–case–control design, since the qualitative comparison between the two analyses would have suffered from a poor statistical power. Second, we were able to match our cases to controls (on sex and the length of ICU stay until HAI onset) and accurately adjust for time at risk, which it is one of the biases of utmost importance to consider when the outcome is HAI onset, given that it is strongly associated with the length of hospitalization [25]. Moreover, by using incidence density sampling with replacement rather than survivor sampling, the OR from our nested case–control study is a direct estimate of the incidence rate ratio from the underlying cohort study [27] and it avoids overestimation of the results coming from a case–case–control design that applies survivor sampling in the presence of an outcome that is not rare (<5%) [28], as was in our population.

As for the results of our analyses, we found that previous exposure to aminoglycosides, linezolid, penicillins, and colistin increased the likelihood of developing an infection sustained by MDR-Kp among ICU patients. Specifically, aminoglycosides were the only agents with consistent results in the two multivariable models, indicating that not only previous exposure but also its duration may play a role in such infections, strongly supporting our findings. Indeed, previous exposures to aminoglycosides have been shown to be associated with increased colonization by MDR-Kp [21], as well as the incidence and proportion of resistant *K. pneumoniae* in a time-series study [14]. Linezolid, the only oxazolidinone prescribed to our patients, has been shown to increase the colonization by ESBL-producing *K. pneumoniae* in the mouse gut [29], while in human patients, it was found to be associated with MDR-Kp colonization or infection at univariate analysis only [21,30,31]. Since the association remained after adjustment for other factors in our study, this could indicate that further research investigating linezolid’s role is needed to assess its association with *K. pneumoniae* infections and rationalize its use in critically ill patients [32]. Indeed, *K. pneumoniae* has intrinsic resistance to linezolid, and the increased association we found is probably mediated by disruption of the linezolid-susceptible microflora and subsequent overgrowth of colonizing ESBL-Kp [29]. The same mechanism has been shown for vancomycin but with a time-dependent effect [29], and the lack of significance for glycopeptides in our analysis could depend on the shortness of its cumulative exposure.

In our analyses, consumption of penicillins combined with beta-lactamase inhibitors resulted in another factor capable of influencing the onset of the aforementioned infections. The finding is consistent with the literature in which treatments with penicillins/beta-lactamase inhibitors appeared associated with the isolation of MDR Gram-negative bacilli, including MDR-Kp [13,16,33,34]. The days of exposure to colistin were associated with MDR-Kp infection in our cumulative exposure model but not in the dichotomous model. This is in line with Mantzarlis et al. [35], who found similar results for the two different ways to account for antibiotic exposure. In addition, we were not able to demonstrate an effect of other antibiotic classes often associated with CR or/ and ESBL Kp in the literature [15], such as carbapenems [16,21,36,37,38], fluoroquinolones [12,13,39], and extended-spectrum cephalosporins [13,14,39]. In our opinion, this may be due to the different study designs or outcomes of these studies, mostly investigating temporal trend association or colonization, together with infections.

While a boosting interaction between carbapenems and fluoroquinolones has been described [16], to the best of our knowledge, there is no report in the literature on a combined effect of penicillins and oxazolidinones (or polymyxins) on the development of infections sustained by MDR-Kp; interestingly, we found that the effect of a combined exposure in the time window before the infection seemed to be sufficient to hinder the selective pressure exerted by individual agents. These findings could highlight the high variability in the results obtained by epidemiological and ecological studies on the capacity of antibiotic pressure to influence the ecology of the pathogen of interest [24]. Hence, more standardized studies are needed to better identify the predictors of antimicrobial infections sustained by multidrug-resistant microorganisms and limit the antibiotic use as much as possible [8]. However, from a stewardship perspective, it is important to mention that all the antibiotic classes individuated as a risk factor for MDR-Kp-sustained infections in our study are clinically useful and sometimes even the last-resort drugs against MDR Gram-negative bacteria in current clinical practice [40,41,42]; for example, the high consumption of aminoglycosides and colistin in our ICU could be related to the high incidence of HAIs caused by *A. baumannii* with an extensively drug-resistant or pandrug-resistant profile registered during surveillance activities, which leaves almost no option to clinicians [43]. Hence, since these findings consistently support a role of aminoglycosides in the development of infections sustained by MDR-Kp, stewardship recommendation on the use of such antibiotics needs to be carefully balanced with their recognized usefulness.

Lastly, although our cases were slightly more compromised than the controls, none of the variables related to the patients’ clinical conditions (e.g., type of admission to the ICU, exposure to mechanical ventilation, preexisting comorbidities, SAPS II) seemed to affect infection onset, in line with the literature [12,16,21,35,44]. Furthermore, the fact that we did not find any relationship with the year of admission may be due to the active surveillance that was carried out in those years in the ICU [43], whose primary purpose was containment of the spread of HAIs and monitoring of infection trends, not to mention the multimodal intervention aimed at improving healthcare worker adherence to standard hygiene precautions that was implemented in the ward in 2017 [45], which may have limited the effects of the hospital environment on HAI onset.

This study had several strengths and limitations. The main strength was that data on antibiotic exposures were collected during the follow-up period in a hospital setting, meaning that distortion of the results due to recall bias or non-compliance was unlikely. Furthermore, by using incidence density sampling to identify controls, we were able to fully represent the underlying pool of time at risk for the cohort members. We also analyzed the effect of prior exposure to antibiotics and the cumulative duration of such exposure. By contrast, as previously discussed, the major limitation was represented by the case–control study design, since in this case, we could not identify risk factors uniquely associated with the resistant phenotype of *K. pneumoniae* [25]; however, given the extremely small number of non-MDR *K. pneumoniae* in our case history, the feasibility of either a case–case–control or a case–control–control study was limited. Second, given the impossibility to match our patients on the length of stay and disease severity simultaneously, and since patients who are hospitalized longer have more opportunities both to receive antibiotics and to acquire a resistant organism and are generally sicker [25], we focused on the length of stay, an important measure of time at risk. That means that we may have not fully accounted for disease severity. However, all the related variables (SAPS II, any preexisting comorbidity, and the type of ICU admission) were included in our multivariable analyses, limiting as much as possible any residual confounding. In addition, a comprehensive past antibiotic history outside the ward was not available; however, the number of cases that developed an infection within a few days after ICU admission was minimal. Furthermore, we considered exposure to more than one antibiotic class irrespective of the administration timing (i.e., we did not consider whether the double exposure was simultaneous or a few days apart). Lastly, we were not able to assess the role of the antibiotic dose on the development of resistance. Such analysis could be an interesting area for future investigations to further study risk factors that may contribute to the onset of infections sustained by *K. pneumoniae*.

## 4. Materials and Methods

### 4.1. Data Collection

This was a nested case–control study that used data on ICU patients retrieved from the active HAI surveillance system conducted from 1 May 2016 to 30 September 2019 in the main ICU of the Umberto I Teaching Hospital of Rome. The detailed methodology of the surveillance system is described elsewhere [43]. Briefly, the diagnostic criteria for the identification of HAIs were based on the National Healthcare Safety Network protocol of the Centers for Disease Control [46], while the protocol of the European Center for Disease Prevention and Control [47] was used to identify BSIs. The surveillance system required that all patients hospitalized in the ICU for at least two consecutive calendar days be monitored until their discharge from the ICU. Among device-related HAIs, the incidence of CRBSIs, VAP, and CAUTIs that occurred after 48 h from device insertion, was registered. The surveillance system also routinely stored data on the incidence of primary BSIs and SSIs that occurred 48 h after ICU admission or within 30 days after surgery, respectively.

A standardized form with four sections was used to collect data systematically. These sections referred to (i) patient demographics and information about hospitalization (date of hospitalization, date of admission to the ICU, type of admission to the ICU, discharge date, status of the patient at discharge, preexisting comorbidities, SAPS II) within 24 h from ICU admission; (ii) exposure to risk factors (information about the start and end dates and duration of the patient’s exposure to urinary catheterization, central venous catheterization, and mechanical ventilation) and whether the device was present within 48 h before the onset of infection; (iii) antibiotic therapy (information about the agent(s) used, route of administration, start and end dates of antibiotic therapy for each agent used); and (iv) diagnosed HAIs and microbiological cultures performed (site of infection, date of HAI onset, and microbiological confirmation (date of sample collection and microorganisms identified, together with their resistance or sensitivity to the various antibiotics tested in the antibiogram)).

We defined as cases patients who developed an HAI sustained by MDR-Kp (i.e., including multidrug resistant, extensively drug resistant, or pandrug resistant, according to the classification of the resistance profile proposed by Magiorakos et al. [48]) over their ICU stay. In the case of multiple infections per individual sustained by MDR-Kp, only the first episode was considered. Each case was matched on sex and the length of ICU stay until HAI onset to three controls randomly selected from at-risk patients using incidence density sampling with replacement. Eligible controls were defined as patients without an infection sustained by MDR-Kp at the time of selection or before, regardless of infections sustained by other microorganisms during their hospitalization (i.e., a control could have become a case by developing an MDR-Kp-sustained infection later).

We considered prior antibiotic exposure as having used any antibiotic agent through systemic administration (i.e., enteral or parenteral) for a different clinical reason than the MDR-Kp infection in the time period from ICU admission to the day before HAI onset (for the cases) or having used any antibiotic agent through systemic administration (i.e., enteral or parenteral) in the time period from ICU admission to the day before they were selected as controls (for the controls). Prior antibiotic exposure was coded as (i) dichotomous (yes/no: yes was assigned in the case of at least two days of prior exposure) or (ii) cumulative (sum of the days of prior exposure).

The institutional ethics board of the Umberto I Teaching Hospital of Rome approved the study protocol (reference number: 6036/2020).

### 4.2. Statistical Analysis

For the purposes of this analysis, only the most frequently prescribed antibiotic agents were used. Agents belonging to the same class were grouped (Supplementary Table S1). Descriptive statistics were obtained using means and standard deviations for continuous variables and proportions for dichotomous and categorical variables. Exposure to the antibiotic classes was analyzed in two ways: First, to provide an overall assessment of the patient’s exposure(s), each exposure was dichotomized (yes vs. no), considering two days of antibiotic administration as a minimum length of exposure. Second, to quantify the cumulative duration of such exposure(s), the days of antibiotic use within each class were summed. As for the presence of preexisting comorbidities (diabetes mellitus, asthma, pulmonary fibrosis, coronary heart disease, chronic kidney disease, chronic liver disease, active cancer, immunodeficiency, and transplant), they were collapsed into one variable with two levels (i.e., having at least one chronic condition vs. having none).

Univariate analysis was performed using the Student t-test for continuous variables and using the chi-square test or the Fisher exact test, if appropriate, for dichotomous variables.

Multivariable conditional logistic regression analyses were performed to estimate odds ratios (ORs) and their 95% confidence interval (CI) of prior exposure to each antibiotic class (yes vs. no, Model 1) or its duration (cumulative days of antibiotic class use, Model 2) and the likelihood of HAIs sustained by MDR-Kp. The effect of exposure to each antibiotic class was adjusted for other antibiotic classes and for non-antibiotic risk factors, as suggested by Schechner et al. [25]. All possible two-way interaction terms between the antibiotic classes were examined by adding them, one at a time, to the main effects model and were tested using a significance level cutoff of 0.05, whereas collinearity was detected using a variance inflation factor cutoff of >2.5. Since days of central venous catheterization, days of urinary catheterization, and days of mechanical ventilation were highly collinear, only the latter was kept for further analyses. As a result, the following variables were used to build the two models: prior exposure to aminoglycosides, prior exposure to carbapenems, prior exposure to extended-spectrum cephalosporines, prior exposure to glycopeptides, prior exposure to daptomycin, prior exposure to linezolid, prior exposure to colistin, prior exposure to penicillins, age (<40 years, 41–50 years, 51–60 years, 60–70 years, >70 years), year of admission (continuous), preexisting comorbidities (yes vs. no), days of mechanical ventilation (continuous), SAPS II (continuous), and type of admission to the ICU (medical, trauma, or post-surgery). All analyses were performed using STATA version 15.1 software (StataCorp LLC, 4905 Lakeway Drive, College Station, TX, USA). The significance level was set to a *p*-value of less than 0.05, and all tests were two-sided.

## 5. Conclusions

Our study showed that particular attention should be drawn to the use of a few antibiotic classes that may play a role in infections sustained by MDR-Kp: specifically, we found that previous exposure to aminoglycosides, linezolid, penicillins, and colistin increases the likelihood of infection onset among ICU patients, together with a hindering effect of a combined exposure to penicillins and linezolid or colistin. These findings suggest the need for conducting more standardized studies in this field to better understand the role of antibiotics in influencing the ecology of *K. pneumoniae*.

## Figures and Tables

**Table 1:** Characteristics of the patients included in the nested case–control study. Results are expressed as number (percentage) or mean ± standard deviation.

	**Cases**	**Controls**	***p*-Value**
Patients	87	261	
Gender (male)	61 (70.1)	183 (70.1)	NA
Age, years	57.5 ± 17.3	56.5 ± 18.4	0.657
Type of admission to the ICU			0.788
Medical	43 (49.4)	124 (47.5)	
Trauma	31 (35.6)	103 (39.5)	
Post-surgery	13 (15.0)	34 (13.0)	
Pre-existing comorbidity	32 (36.8)	80 (30.7)	0.292
Diabetes mellitus	17 (19.5)	55 (21.0)	0.879
Asthma	3 (3.5)	2 (0.8)	0.102
Pulmonary fibrosis	0 (0.0)	1 (0.4)	1.000
Coronary heart disease	8 (9.2)	13 (5.0)	0.153
Chronic kidney disease	7 (8.1)	8 (3.1)	**0.048**
Chronic liver disease	0 (0.0)	1 (0.4)	1.000
Active cancer	2 (2.3)	10 (3.8)	0.737
Immunodeficiency	1 (1.2)	0 (0.0)	0.250
Transplant	1 (1.2)	0 (0.0)	0.250
Year of admission			0.269
2016	15 (17.2)	48 (18.4)	
2017	29 (33.3)	65 (24.9)	
2018	26 (29.9)	73 (28.0)	
2019	17 (19.5)	75 (28.7)	
SAPS II score	42.8 ± 13.7	40.6 ± 14.1	0.195
Urinary catheter, days	23.7 ± 17.6	22.7 ± 17.8	0.649
Central venous catheter, days	23.3 ± 17.7	21.7 ± 17.0	0.442
Mechanical ventilation, days	19.7 ± 13.4	18.8 ± 16.3	0.642

ICU: intensive care unit; SAPS: Simplified Acute Physiology Score. Significant *p*-values are indicated in bold.

**Table 2:** Prior antibiotic exposure expressed as dichotomous (number, percentage) or cumulative (in days, mean ± standard deviation) comparing cases and controls. Cumulative exposure reports the mean days of antibiotic use for each group, considering both exposed and unexposed patients.

**Antibiotic Class**	**Dichotomous Exposure** **(Yes, at Least Two Days)**			**Cumulative Exposure** **(Days)**		
	**Cases**	**Controls**	***p*** **-Value**	**Cases**	**Controls**	***p*** **-Value**
Carbapenems	84 (96.6)	242 (92.7)	0.203	33.3 ± 36.5	29.6 ± 34.3	0.390
Penicillins	68 (78.2)	191 (73.2)	0.356	7.3 ± 6.5	6.8 ± 6.2	0.484
Polymyxins	44 (50.6)	117 (44.8)	0.352	10.1 ± 17.0	8.0 ± 14.1	0.260
Extended-spectrum cephalosporins	41 (47.1)	112 (42.9)	0.493	3.7 ± 6.9	4.2 ± 7.6	0.587
Glycopeptides	29 (33.3)	87 (33.3)	1.000	2.9 ± 5.3	3.0 ± 5.3	0.812
Oxazolidinones	33 (37.9)	77 (29.5)	0.143	2.4 ± 4.2	1.8 ± 3.8	0.200
Aminoglycosides	24 (27.6)	41 (15.7)	0.014	1.8 ± 3.7	1.1 ± 2.9	0.074
Lipopeptides	22 (25.3)	50 (19.2)	0.222	1.8 ± 4.1	1.5 ± 3.8	0.528

Polymyxins (colistin). Oxazolidinones (linezolid). Lipopeptides (daptomycin).

**Table 3:** Types of healthcare-associated infections (HAIs) registered by the active surveillance system among the cases and controls. Infections among the cases are sustained by multidrug-resistant *Klebsiella pneumoniae*, while infections among the controls are sustained by microorganisms other than multidrug-resistant *Klebsiella pneumoniae.* Results are expressed as numbers (percentages).

	**Cases (*n* = 87)**	**Controls (*n* = 261)**
Device-related HAI		
VAP	13 (15.0)	11 (4.2)
CRBSI	11 (12.6)	4 (1.5)
CAUTI	28 (32.2)	8 (3.1)
BSI	31 (35.6)	8 (3.1)
Surgical site infection	4 (4.6)	1 (0.4)
Without HAI	0 (0.0)	229 (87.7)

VAP: ventilator-associated pneumonia; CRBSI: catheter-related bloodstream infection; CAUTI: catheter-associated urinary tract infection; BSI: bloodstream infection.

**Table 4:** Multivariable conditional logistic regression models for developing healthcare-associated infections sustained by multidrug-resistant *Klebsiella pneumoniae* (Model 1: prior exposure to an antibiotic class is coded as dichotomous (yes vs. no); Model 2: prior exposure to an antibiotic class is coded as continuous (days of cumulative exposure)).

	**Model 1**		**Model 2**	
	**OR (95% CI)**	***p*-Value**	**OR (95% CI)**	***p*-Value**
Age (category)	1.00 (0.81–1.24)	0.971	0.94 (0.76–1.17)	0.587
Aminoglycosides	**2.27 (1.08–4.76)**	**0.030**	**1.15 (1.03–1.29)**	**0.015**
Carbapenems	0.83 (0.17–4.12)	0.824	0.98 (0.95–1.00)	0.094
Extended-spectrum cephalosporins	1.40 (0.74–2.62)	0.299	0.99 (0.94–1.05)	0.781
Glycopeptides	0.93 (0.51–1.70)	0.824	1.01 (0.95–1.07)	0.847
Lipopeptide (daptomycin)	1.31 (0.65–2.63)	0.455	1.01 (0.94–1.09)	0.747
Oxazolidinones (linezolid)	**4.64 (1.32–16.27)**	**0.016**	1.07 (1.00–1.16)	0.055
Penicillins	2.32 (0.84–6.38)	0.103	**1.08 (1.01–1.16)**	**0.018**
Polymyxins (colistin)	1.08 (0.85–3.83)	0.811	**1.07 (1.02–1.14)**	**0.013**
Type of admission to the ICU				
Medical	Ref.	-	Ref.	-
Trauma	0.90 (0.45–1.80)	0.776	0.87 (0.43–1.76)	0.692
Post-surgery	1.20 (0.52–2.78)	0.674	1.33 (0.58–3.05)	0.499
Mechanical ventilation, days	1.02 (0.99–1.05)	0.197	1.02 (0.99–1.06)	0.209
SAPS II	1.01 (0.99–1.05)	0.341	1.01 (1.00–1.04)	0.138
Pre-existing comorbidity	1.31 (0.68–2.51)	0.417	1.37 (0.72–2.61)	0.337
Year of admission	0.84 (0.64–1.11)	0.220	0.89 (0.69–1.15)	0.374
Oxazolidinones (linezolid)*Penicillins	**0.22 (0.05–0.88)**	**0.032**	-	-
Penicillins*Polymyxins(colistin)	-	-	**0.996 (0.993–0.999)**	**0.012**

OR: odds ratio; CI: confidence interval; ICU: intensive care unit; SAPS: Simplified Acute Physiology Score. * Interaction term. Ref: reference category. Statistically significant ORs are indicated in bold.

## Data Availability

Data available on reasonable request due to privacy reasons.

## Supplementary Materials

The following are available online at https://www.mdpi.com/2079-6382/10/3/302/s1, Table S1: Classification of antibiotic agents into their respective antibiotic classes.
