# Supplementary material for: Prior Antibiotic Therapy and the Onset of Healthcare-Associated Infections Sustained by Multidrug-Resistant Klebsiella pneumoniae in Intensive Care Unit Patients: A Nested Case–Control Study
Source: Antibiotics (Basel). 2021 Mar 15;10(3):302. doi: 10.3390/antibiotics10030302 (PMC8000440; doi:10.3390/antibiotics10030302)
Supplement: Supplementary file 1 [file antibiotics-10-00302-s001.pdf]

antibiotics

MDPI

Article

Prior Antibiotic Therapy and the Onset of Healthcare-Associated Infections Sustained by Multidrug-Resistant *Klebsiella pneumoniae* in Intensive Care Unit Patients: A Nested Case–Control Study

Giuseppe Migliara [1,§,*], Valentina Baccolini [1,§], Claudia Isonne [1], Sara Cianfanelli [1], Carolina Di Paolo [1], Annamaria Mele [1], Lorenza Lia [1], Angelo Nardi [1], Carla Salerno [1], Susanna Caminada [1], Vittoria Cammalleri [1], Francesco Alessandri [2], Guglielmo Tellan [2,3], Giancarlo Ceccarelli [1], Mario Venditti [1], Francesco Pugliese [2,4], Carolina Marzuillo [1], Corrado De Vito [1], Maria De Giusti [1], and Paolo Villari [1]

§ These authors contributed equally

1 Department of Public Health and Infectious Diseases, Sapienza University of Rome, 00185 Rome, Italy; valentina.baccolini@uniroma1.it (V.B.); claudia.isonne@uniroma1.it (C.I.); sara.cianfanelli@uniroma1.it (Sara Cianfanelli); carolina.dipaolo@uniroma1.it (C.D.P.); annamaria.mele@uniroma1.it (A.M.); lorenza.lia@uniroma1.it (L.L.); angelo.nardi@uniroma1.it (A.N.); carla.salerno@uniroma1.it (C.S.); susanna.caminada@uniroma1.it (Susanna Caminada); vittoria.cammalleri@uniroma1.it (V.C.); giancarlo.ceccarelli@uniroma1.it (G.C.); mario.venditti@uniroma1.it (M.V.); carolina.marzuillo@uniroma1.it (C.M.); corrado.devito@uniroma1.it (C.D.V.); maria.degiusti@uniroma1.it (M.D.G.); paolo.villari@uniroma1.it (P.V.)

2 Department of Anesthesia and Intensive Care Medicine Azienda, Universitaria-Ospedaliera Policlinico Umberto I, 00185 Rome, Italy; guglielmo.tellan@uniroma1.it (G.T.); f.pugliese@uniroma1.it (F.P.)

3 Department of Internal, Anesthesiological and Cardiovascular Clinical Sciences, Sapienza University of Rome, 00185 Rome, Italy

4 Department of General and Specialist Surgery "P. Stefanini", Sapienza University of Rome, Rome, Italy

* Correspondence: giuseppe.migliara@uniroma1.it; Tel: +39-06-4991-4886

Table S1. Classification of antibiotic agents into their respective antibiotic class.

Antibiotic Class	Antibiotic Agent
Aminoglycosides	Gentamicin
	Amikacin
	Paromomycin
Carbapenems	Ertapenem
	Imipenem
	Meropenem
Extended spectrum cephalosporines	Cefotaxime
	Ceftazidime
	Cefepime
	Ceftriaxone
	Ceftazidime/Avibactam
	Cefoperazone
	Ceftolozane/Tazobactam
Glycopeptides	Vancomycin
	Teicoplanin
Lipopeptide	Daptomycin
Oxazolidinones	Linezolid
Penicillin	Amoxicillin/Clavulanic acid
	Ampicillin/Sulbactam
	Piperacillin/Tazobactam
Polymyxins	Colistin
